# Characterizing species-specific metabolic signatures in vaginal microbiota across planktonic and biofilm states

**DOI:** 10.1016/j.bioflm.2025.100330

**Published:** 2025-11-13

**Authors:** Smrutiti Jena, Damilola Lawore, Leopold N. Green, Douglas K. Brubaker

**Affiliations:** a Weldon School of Biomedical Engineering, Purdue University, West Lafayette, IN, USA; b Center for Global Health and Disease, Department of Pathology, Case Western Reserve University, Cleveland, OH, USA; c The Blood Heart Lung Immunology Research Center, Case Western Reserve University, University Hospitals of Cleveland, Cleveland, OH, USA

**Keywords:** Bacterial vaginosis, Biofilm, Metabolomics, Metabolic pathway, Metabolites, *L. crispatus*, *G. vaginalis*

## Abstract

Bacterial vaginosis (BV) affects about 29 % of U.S. women, with higher rates in some groups and up to 50–69 % recurrence within a year. It increases the risk of STIs, pregnancy complications, and can cause significant discomfort and reduced quality of life. Prior studies mapped vaginal metabolomes, but individual microbial metabolite signatures remain poorly understood. Given that biofilms exhibit distinct metabolic requirements compared to planktonic cultures, analyzing biofilm vs. planktonic culture metabolites with their unique metabolic needs may uncover novel treatment targets. This study provides a comprehensive metabolomic comparison of key vaginal microbes—*Lactobacillus crispatus, Gardnerella vaginalis*, and *Lactobacillus iners* under both planktonic and biofilm conditions. Our analysis showed that metabolite production and consumption vary by microbe and growth mode. *G. vaginalis* formed biofilms in multiple media, with limited shared metabolic pathways between its biofilm types, indicating unique metabolic processes. Despite L. *crispatus* suspension and biofilm cultures sharing 142 consumed and 104 produced metabolites, the biofilm culture demonstrated a remarkable metabolic shift. Comparing all three species, we observed convergence in nutrient utilization, but divergence in metabolic outputs reflecting growth-specific adaptations, highlighting the importance of considering microbial state in vaginal microbiome studies.

## 1 Introduction

The vaginal microbiota was first described in 1892 by Albert Döderlein as a population mainly of Gram-positive bacilli, now identified as Lactobacillus species originating from the gut [1]. The evolution of this distinctive vaginal microbiome is underpinned by two evolutionary hypotheses: the “disease risk hypothesis" and the “obstetric protection hypothesis". These theories propose that the human vagina is predominantly populated by protective Lactobacillus species due to increased susceptibility to sexually transmitted diseases and higher risks of pregnancy- and childbirth-associated microbial complications [2].

Bacterial vaginosis (BV) is a vaginal disorder whose presentation ranges from asymptomatic cases to those presenting with unusual odor, discharge, and irritation. BV occurs when the microbial community shift from Lactobacillus spp. dominated to anaerobic bacteria overgrowth like *Gardnerella vaginalis* [3]. BV increases the risk of acquiring STIs—including gonorrhea, chlamydia, trichomoniasis, herpes simplex virus, HPV, and HIV—and is linked to pelvic inflammatory disease, endometritis, chorioamnionitis, and amniotic fluid infection. In pregnancy, BV is associated with adverse outcomes such as preterm premature rupture of membranes, preterm labor, and preterm birth [[4], [5], [6], [7], [8], [9]].

BV is marked by a polymicrobial biofilm on the vaginal epithelium, lacking Lactobacilli. These biofilms consist of bacteria encased in a self-produced matrix, adhering to surfaces and each other. [[10], [11], [12]]. Microorganisms within biofilms exhibit distinct behavior compared to planktonic cells, making them more resilient against conventional antimicrobial treatments and enabling evasion of the host's immune response [13,14]. According to Swidsinski, this resistance is why biofilms of *G. vaginalis* in women with BV cannot be eradicated using traditional therapies involving moxifloxacin and metronidazole [15]. Thus, biofilms may contribute to persistent, slow advancing chronic infections. Metabolites facilitate interspecies metabolic collaboration in bacterial communities, exemplified by oxygen gradients in biofilms enabling anaerobic bacteria to thrive alongside aerobes. [[16], [17], [18]]. There are studies that establish cooperative and syntrophic interactions between microbes through metabolic exchanges in a certain niche significantly dictate nutritional quality and habitants of the community [[19], [20], [21]]. Metabolites play a significant role in fostering interkingdom symbiotic relationships, encompassing mutualistic, commensal, and parasitic interactions. Detection of metabolites by one type of host cell can serve to communicate with other cell types within the host, facilitating the coordination of host responses both locally and systemically [22,23].

Recent technological advancements have enabled the analysis of metabolome across various cell types. Here, we generated untargeted metabolomics data from organisms of significance in the vaginal microbiome, with a focus on species associated with the etiology of BV to characterize the differential production or consumption of metabolites across species and discrete metabolic growth conditions like suspension and biofilm cultures.

## 2 Results

### 2.1 Certain metabolites are uniquely generated or consumed in the suspension culture of vaginal microbes

**Fig. 1:**
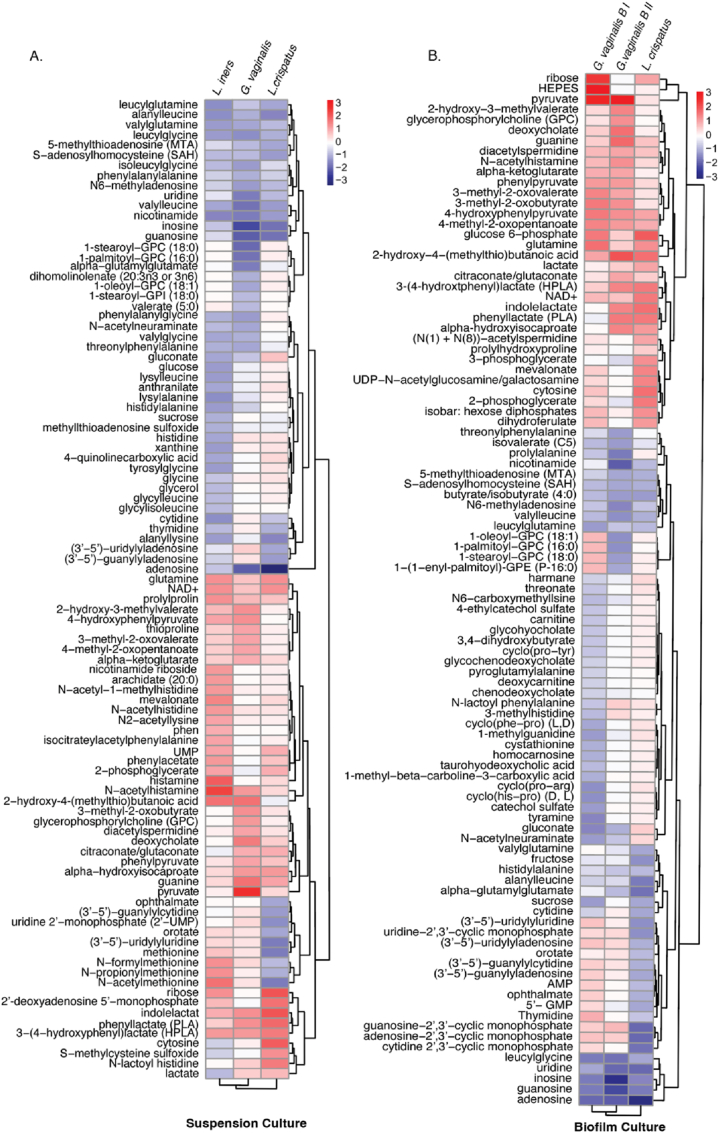
Metabolites generated or consumed by suspension and biofilm cultures across microbes show nutrient-modulated diverse profile. Heatmaps showing differential abundance of metabolites in (A) suspension cultures of *L. crispatus, L. iners* and *G. vaginalis* mapped on log2 (blank media normalized) value and (B) metabolite profile of biofilm culture of *L. crispatus* and both types of *G. vaginalis* biofilms mapped on log2 (blank media normalized) value. *G. vaginalis* B I: Biofilm type I grown in NYCIII media and *G. vaginalis* B II: Biofilm type II grown in supplemented BHI media (sBHI). Red represents produced metabolites, while blue denotes consumed metabolites. (For interpretation of the references to colour in this figure legend, the reader is referred to the Web version of this article.)

We selected metabolites with p < 0.1 in comparison with media and from there we chose a threshold of log2 FC ≥ 4 or ≤ −4 that allowed interpretable visualization of the most prominent metabolites in the heatmap (Fig. 1A & B). Our analysis revealed distinct patterns in the production or consumption of specific metabolites among suspension cultures of *L. crispatus, G. vaginalis* and *L. iners*, as illustrated in Fig. 1A. In this representation, blue indicates metabolites consumed, while red indicates those produced, with intensity reflecting the extent of production or consumption. For example, Gluconate was produced by *L. crispatus* and consumed by both *G. vaginalis* and *L. iners*. Adenosine, though consumed by all, was notably more heavily utilized by *L. crispatus*. Metabolites such as glutamine, NAD+, uridine monophosphate, 2-phosphoglycerate, and ribose were exclusively produced by both Lactobacillus species. However, there were metabolites like N-formyl methionine, N-propionylmethionine, and N-acetyl methionine that were produced by *L. iners* and consumed by *L. crispatus*. Similarly, cytosine and lactate were produced by *L. crispatus* and consumed by *L. iners*. Histamine was found to be significantly and exclusively produced by *L. iners*. Pyruvate and deoxycholate were notably more abundantly produced by *G. vaginalis* compared to the other two microbes (Fig. 1A).

### 2.2 Consumption of metabolite depends on the substrate availability that regulates the composition of produced metabolites in biofilm cultures

Even though the media (MRS) used for both suspension and biofilm cultures of *L. crispatus* remained consistent, noticeable differences were observed in the consumption and production patterns of metabolites across these cultures. Similar distinctions were evident in the suspension and biofilm type I (grown in NYCIII media) of *G. vaginalis*. Furthermore, the metabolite profiles differed between type I and type II (grown in supplemented BHI; details in methods section) biofilms of *G. vaginalis*, indicating distinct utilization and production of metabolites in biofilm culture modes, influenced by available substrates (Fig. 1B). The L. *iners* strain (AB107) used in this study does not produce biofilm. Therefore, not included in the biofilm metabolomics comparisons [24].

*L. crispatus* was the exclusive producer of gluconate, N-acetylneuraminate, and harmane, while both types of *G. vaginalis* biofilms consumed them. Pyruvate, cGMP, and cAMP were solely produced by *G. vaginalis* biofilms, with the latter two being consumed by *L. crispatus*. Notably, in the case of *G. vaginalis* biofilm type II, 1-oleoyl GPC, 1-palmitoyl GPC, 1-stearoyl GPC, and 1−(1−enyl-palmitoyl)-GPE were produced, but in type I, they were consumed. Ribose and HEPES were significantly produced by type II biofilms but not by type I (Fig. 1B).

Enriched metabolite pathways identified from *G. vaginalis* cultures reveal greater similarity between suspension and biofilm type I, while highlighting a significant metabolic distinction between biofilm types I and II.

**Fig. 2:**
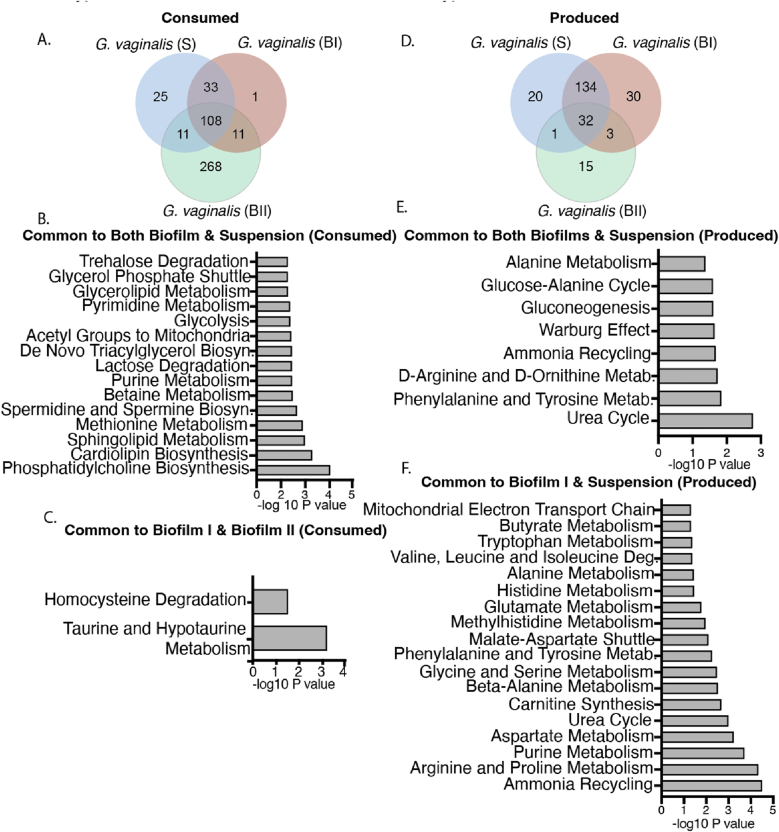
Shared metabolites across culture types of *G. vaginalis* indicate more similarity between suspension and biofilm type I and distinction between the two biofilm types. Intra-strain comparison of metabolite distribution and enrichment pathways corresponding to culture conditions of *G. vaginalis*. (A) Venn diagram showing distribution of consumed metabolites among suspension and biofilm cultures, (B) Common significant pathways for all biofilm and suspension consumed metabolites, (C) Pathways significant for both biofilm type I and biofilm type II based on consumed metabolites, (D) Venn diagram showing distribution of produced metabolites among suspension and biofilm cultures,(E) Common significant pathways for biofilm and suspension produced metabolites, (F) Common significant pathways for suspension and biofilm I produced metabolites. Included pathways with p value ≤ 0.059 and -log10 p value was used to plot the graphs. S: Suspension, BI: Biofilm type I, BII: Biofilm type II, Metab.: metabolism.

A total of 108 metabolites were commonly consumed, while 32 were produced across all forms of *G. vaginalis* culture (Fig. 2A–D). The commonly consumed metabolites across all growth modes were associated with 18 significant metabolic pathways, with phosphatidylcholine biosynthesis emerging as the most prominent, followed closely by the cardiolipin biosynthesis pathway. (Fig. 2B). The 25 metabolites, specifically consumed by suspension culture but none of the biofilm types belonged only either to bile acid biosynthesis or thiamine metabolism (Data not shown). Eleven metabolites were commonly consumed by both biofilm types, primarily associated with taurine and hypotaurine metabolism, followed by homocysteine degradation (Fig. 2C). This minimal overlap highlights the metabolic adaptability and diversity of *G. vaginalis* during biofilm formation in response to nutrient availability, demonstrating that the organism can adopt entirely distinct metabolic pathways based on the resources at its disposal.

In terms of commonly produced metabolites, suspension cultures and biofilm type I shared 134 byproducts, while suspension cultures and biofilm type II shared only one. This indicates that biofilm type I is more similar to suspension culture in its metabolic profile compared to biofilm type II (Fig. 2D). Also, only three commonly produced metabolites were shared between the two biofilm types, and these did not correspond to any specific significant pathway. This underscore distinct metabolic profiles that differentiate the two biofilm types. Among the enriched pathways associated with the produced metabolites, both suspension and type I biofilm cultures were linked to 18 common metabolic pathways, with ammonia recycling identified as the most significant (Fig. 2F). However, with the inclusion of biofilm type II, urea cycle emerged as most predominant common pathway (Fig. 2E). This suggests that biofilm type II is less efficient in conserving and reusing nitrogen compared to suspension cultures and biofilm type I. Pathways like d-ornithine metabolism, warburg effect and gluconeogenesis were only noticed when there was inclusion of biofilm type II along with suspension and type I biofilm (Fig. 2E), (Supplementary Table 1).

### 2.3 Metabolites produced exclusively by suspension or biofilm cultures of *L. crispatus* belong to significantly different metabolic pathways

**Fig. 3:**
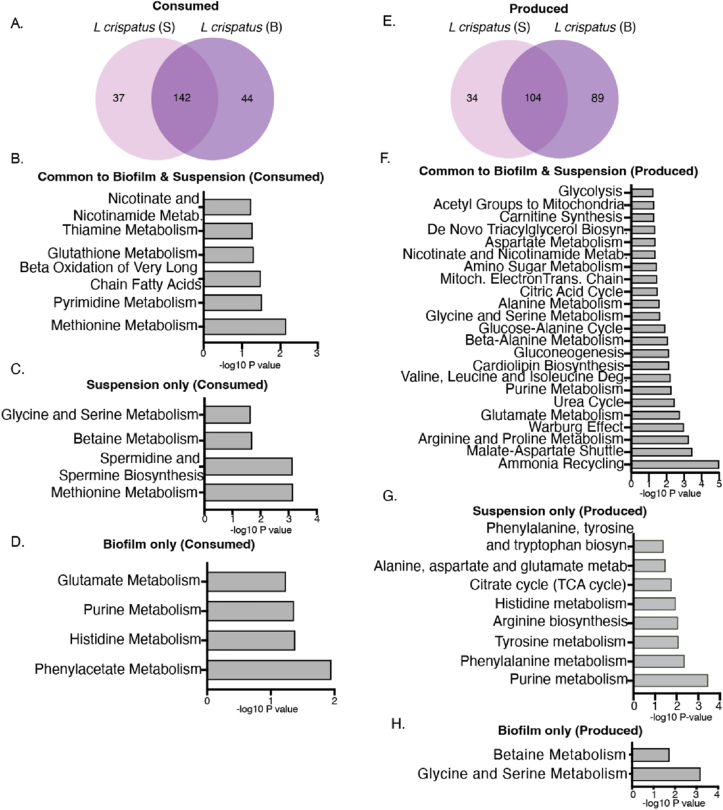
Suspension and biofilm populations of *L. crispatus* exhibit distinct metabolic profiles and have significant differences in key biochemical pathways. Intra-strain comparison of metabolite distribution and enrichment pathways corresponding to culture conditions of *L. crispatus*.(A) Venn diagram showing distribution of consumed metabolites among suspension and biofilm cultures, (B) significant pathways for biofilm and suspension shared consumed metabolites, (C) key metabolic pathways uniquely associated with suspension consumed metabolites, (D) key metabolic pathways uniquely associated with biofilm consumed metabolites, (E) Venn diagram showing distribution of produced metabolites among suspension and biofilm cultures, (F) significant pathways for biofilm and suspension shared produced metabolites, (G) key metabolic pathways uniquely associated with suspension produced metabolites, (H) key metabolic pathways uniquely associated with biofilm produced metabolites. Included pathways with p value ≤ 0.059 and -log10 p value was used to plot the graphs. S: Suspension, B: Biofilm, Metab.: metabolism, Deg.: Degradation, Biosyn.: Biosynthesis.

We discovered that *L. crispatus* suspension and biofilm cultures commonly consumed 142 metabolites and produced 104 metabolites, respectively (Fig. 3A–E). The metabolites commonly consumed by both cultures were associated with six metabolic pathways, with methionine metabolism emerging as the most significantly abundant (Fig. 3B). Metabolites exclusively consumed by suspension cultures also predominantly belonged to the methionine metabolism pathway, closely followed by spermidine and spermine biosynthesis (Fig. 3C). In contrast, the metabolic profile of biofilm-exclusive consumed metabolites revealed a distinct set of pathways, most notably phenylacetate metabolism, followed by histidine metabolism, purine metabolism, and glutamate metabolism (Fig. 3D).

Metabolites commonly produced by both cultures were significantly associated with 22 metabolic pathways, with ammonia recycling emerging as the most prominent. This set of pathways was notably distinct from those observed in either culture individually (Fig. 3F). The 34 metabolites exclusive to suspension culture highlighted purine metabolism as the most significant, followed by phenylalanine and tyrosine metabolism, arginine biosynthesis, histidine metabolism, and the TCA cycle (Fig. 3G). In contrast, the biofilm culture of *L. crispatus* diverged from its suspension counterpart, primarily in glycine and serine metabolism and betaine metabolism pathways (Fig. 3H), (Supplementary Table 1).

Commonly consumed metabolites in suspension or biofilm cultures of *L. crispatus, L. iners*, and *G. vaginalis* are predominantly associated with similar metabolic pathways, unlike produced metabolites.

**Fig. 4:**
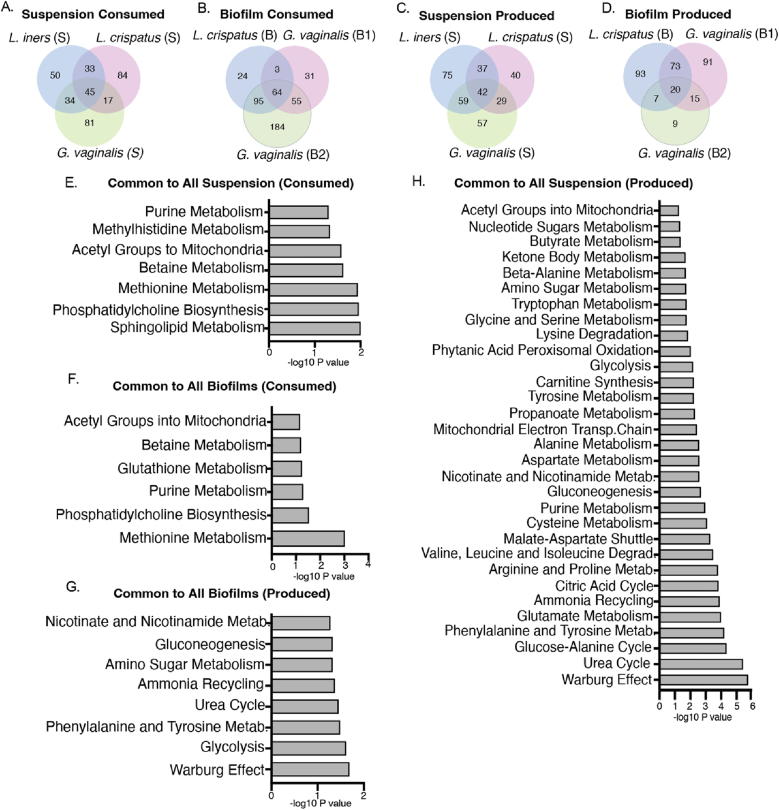
Biofilm produced metabolites belonged to more distinct pathways compared to suspension produced metabolites unlike consumed metabolites, that shows relatively more similar pathways between the two culture types Inter-microbial comparison of metabolite enrichment pathways of *L. crispatus, G. vaginalis*, *L. iners* suspension cultures and *L. crispatus* and *G. vaginalis* biofilm cultures (A–D) Venn diagrams showing distribution of metabolites common or exclusive for suspension and biofilm cultures, (E) significant pathways for all suspension consumed shared metabolites, (F) significant pathways for all biofilm consumed shared metabolites (G) significant pathways for all biofilm produced shared metabolites (H) significant pathways for all suspension produced shared metabolites. Included pathways with p value ≤ 0.059 and -log10 p value was used to plot the graphs. S: Suspension, B: Biofilm, B1: Biofilm type I, B2: Biofilm type II, metab.: Metabolism, Degrad.: Degradation.

Our metabolomic analysis revealed 45 metabolites commonly consumed in suspension cultures of *L. crispatus, L. iners,* and *G. vaginalis* and 64 metabolites in biofilm cultures of *L. crispatus*, and both types of *G. vaginalis* biofilms (Fig. 4A and B). Pathway enrichment analysis of these shared “consumed” metabolites in both culture types display methionine metabolism, phosphatidylcholine biosynthesis, purine metabolism, betaine metabolism, and mitochondrial acetyl group transfer as common pathways followed (Fig. 4E and F). Suspension cultures differ from biofilm only by sphingolipid and methylhistidine metabolism pathways (Fig. 4E). Glutathione metabolism emerged as the sole exclusive pathway enriched in biofilm cultures (Fig. 4F). Conversely, the “produced” metabolites shared among suspension cultures were associated with distinct pathways compared to those produced by biofilm cultures (Fig. 4G and H). Metabolites exclusively consumed by *L. iners* predominantly belonged to amino acid pathways, including glycine, serine, glutathione, and alanine metabolism, coupled with ammonia recycling processes. (Supplementary Fig. 1a).

Pathway enrichment analysis of the 42 suspension-produced common metabolites (Figs. 4C) and 20 biofilm-produced metabolites (Fig. 4D) revealed 31 and 8 enriched pathways followed respectively (Fig. 4G and H). Suspension cultures followed 23 unique pathways than biofilm cultures (Fig. 4G and H). This distribution highlights the metabolic divergence between suspension and biofilm growth modes with respect to produced metabolites unlike consumed metabolites that show more similarity. *L. iners* produces 75 unique metabolites largely associated with eight metabolic pathways, with homocysteine degradation being the most prominent, followed by taurine and hypotaurine metabolism (Supplementary Fig. 1b), (Supplementary Table 1).

Certain metabolically essential host compounds were differentially produced or consumed by planktonic and/or biofilm cultures of key vaginal microbiota.

**Fig. 5:**
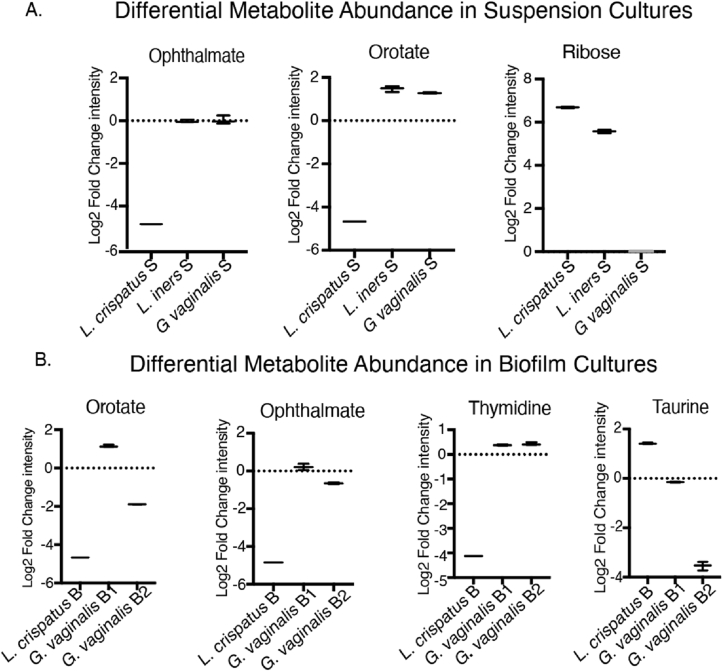
Differential abundance of representative key physiological metabolites among *L. crispatus, G. vaginalis,* and *L. iners* Cultures (A) Box plots of selected metabolites in suspension culture showing differential abundance between microbes, (B) Box plots of selected metabolites in biofilm culture showing differential abundance between microbes. Biological triplicate values were used to plot the graph. Log 2 fold change <0: Consumed and >0: Produced. S: Suspension, B: Biofilm, B1: Biofilm type I, B2: Biofilm type II.

Ophthalmate, accumulation of which is a biomarker of oxidative stress, was found to be significantly consumed by *L. crispatus* suspension and biofilm cultures only. Orotate, a precursor for pyrimidine biosynthesis was consumed by both suspension and biofilm cultures of *L. crispatus* and *G. vaginalis* biofilm type II. This compound was seen to be minimally produced by *L. iners* and *G. vaginalis* suspension and *G. vaginalis* biofilm type I (Fig. 5A and B). Ribose was significantly produced by suspension culture of both the Lactobacillus spp. in this study but neither produced nor consumed by *G. vaginalis* (Fig. 5A). Thymidine, crucial for DNA synthesis and repair, was exclusively utilized by *L. crispatus* biofilm (Fig. 5B). Taurine, a sulfonic acid with critical functions in the central nervous system, exhibited contrasting metabolic patterns among vaginal microbiota. It was synthesized by *L. crispatus* biofilms while being significantly catabolized by *G. vaginalis* type II biofilms. (Fig. 5B).

## 3 Discussions

In recent years, metabolomics has emerged as a potent tool, complementing other 'omic' studies and delving into the extensive metabolic diversity observed in prokaryotes [[25], [26], [27]]. Although metabolomics is viewed as a promising approach for BV identification, no study has comprehensively compared metabolite profiles from pure vaginal microbial cultures, nor has any investigated metabolic differences between their planktonic and biofilm forms [28].

This study demonstrates that vaginal microbes show distinct metabolic profiles in planktonic versus biofilm states, reflecting metabolic diversity within biofilms driven by gradients of oxygen, pH, and nutrients.[29]. The variation in metabolites from *G. vaginalis* biofilms under different media highlights nutrient availability as a key factor influencing biofilm metabolism and development[30].

In this study, both *L. crispatus* and *L. iners* were prominent producers of glutamine, a compound known to influence gut glutathione production, lymphocyte proliferation, and the secretion of certain cytokines [31,32]. Lactobacillus species, especially L. crispatus, produce NAD + at higher levels in biofilm cultures. While research linking NAD+ in the vaginal microbiome to BV or fertility is sparse, gut studies suggest microbial NAD + production supports barrier function and immune health. [33,34]. We noticed gluconate production exclusively in *L. crispatus,* while both types of *G. vaginalis* biofilm consumed it. This aligns with previous reports indicating that pathogenic bacteria utilize and require gluconate for virulence and antibiotic resistance [35,36]. Furthermore, *L. crispatus*, was found to produce kynurenine, a signature compound in a healthy vaginal environment with higher levels in biofilm as compared to suspension culture [37]. Substantial consumption of kynurenine by *G. vaginalis* in both suspension and biofilm forms, as well as by *L. iners* in suspension cultures was observed. In this study, *L. iners* were identified as the only significant producers of histamine that plays significant roles in reproductive physiology, including ovulation, embryo implantation, placental barrier regulation, lactation, and uterine contractions. Additionally, histamine dysregulation is implicated in pregnancy complications like pre-eclampsia and preterm birth [[38], [39], [40]]. *L. iners* produced alternate lactate derivatives such as phenyllactate, imidazole lactate, and indolelactate. In nutrient-rich, low-glucose BHI media as compared to MRS, *L. iners* likely uses amino acid catabolism—producing phenyllactate from phenylalanine, imidazole lactate from l-histidine, and indolelactate from tryptophan.

Metabolomic pathway analysis of consumed metabolites from *G. vaginalis* planktonic and biofilm cultures identified phosphatidylcholine (ChoP) biosynthesis as a key shared metabolic pathway (p = 0.000976). ChoP has been implicated in bacterial virulence and pathogenicity through its role in modulating host immune responses [41]. Lecithinase, a virulence factor of *G. vaginalis* hydrolyzes ChoP, leading to reproductive cell and tissue damage [42]. These findings highlight the complex interplay between *G. vaginalis* metabolism, virulence factors, and host interactions in the context of reproductive tract infections. Besides, we found that *G. vaginalis* consumes metabolites involved in cardiolipin biosynthesis pathway (p = 0.00532). This again, draws parallels to the intestinal pathogen *Shigella flexneri,* suggesting that cardiolipin might play a similar role in enhancing *G. vaginalis* virulence, possibly by supporting membrane integrity, protein localization, or other virulence-associated functions [43]. Our metabolomic analysis revealed contrasting patterns in taurine metabolism between *G. vaginalis* and *L. crispatus*. Both biofilm types of *G. vaginalis* consumed metabolites associated with the taurine metabolism pathway, whereas *L. crispatus* biofilms exhibited notable production of taurine. The role of taurine in vaginal health remains understudied, despite its significant functions in other physiological systems. [44,45]. The phenylacetate metabolism pathway, known to help bacteria like Acinetobacter manage stress responses including acid stress and antibiotic exposure, was identified as a significant pathway (p = 0.011) uniquely active in *L. crispatus* biofilm cultures in this study [46]. Several bacteria, including Lactobacillus species, have been reported to use glutamate metabolism for acid stress tolerance [47]. In this study, we found significant expression of glutamate metabolism in all suspension cultures (p = 0.0001), *L. crispatus* biofilm (p = 0.05), and both biofilm and suspension cultures of *G. vaginalis* (p = 0.01), indicating their adaptation to the acidic niche of the lower reproductive tract. We noticed striking differences in the metabolite profiles of the two types of *G. vaginalis* biofilms, with only two common metabolic pathways identified based on consumed metabolites. This observation highlights metabolic plasticity as an important adaptive strategy, allowing *G. vaginalis* to thrive in diverse nutrient environments. Such metabolic adaptability may contribute to the persistence and virulence of *G. vaginalis* in different host microenvironments. Understanding these metabolic adaptations could provide insights into *G. vaginalis* biofilm formation mechanisms.

This study represents a pioneering effort towards elucidating the specific growth requirements of these microbes that are significant to women's health. Moreover, our data revealed a differential metabolite profile associated with critical metabolic pathways in biofilms of significant vaginal microbes compared to their planktonic cultures. Specifically, we identified metabolites exclusively required for the growth of *L. crispatus*, which could be leveraged to enhance the growth and stability of this beneficial microbe, thereby promoting a healthy vaginal environment. However, untargeted metabolomics can sometimes have sensitivity issues and technical limitations inherent in the analytical platform. Therefore, a technical dropout on lactate levels in *L. iners* gave the appearance of *L iners* consuming lactate when compared to its control. Also, acetic acid a major product generated by *G. vaginalis*, missed the panel of detected metabolites in all culture conditions. The use of synthetic media and limited oxygen availability during culture growth reduces the extent to which the experimental conditions mimic the vaginal microenvironment. Additionally, employing only a single strain per organism demonstrates the need for broader studies incorporating clinical vaginal isolates to more accurately assess the significance of the identified metabolites and pathways for their significance. Finally, these bacteria exist in organized communities *in vivo* and their interactions with one another as well as the host can shape their metabolic outputs. Future studies systematically comparing culture conditions to *in vivo* metabolomics from patients would be beneficial for enhancing the translational relevance of these data.

## 4 Conclusion

This study represents a pioneering effort towards elucidating the specific growth requirements of these microbes that are significant to women's health. Moreover, our data revealed a differential metabolite profile associated with critical metabolic pathways in biofilms of significant vaginal microbes compared to their planktonic cultures. Specifically, we identified metabolites exclusively required for the growth of *L. crispatus*, which could be leveraged to enhance the growth and stability of this beneficial microbe. Comparison of significant metabolic pathway differences between planktonic and biofilm modes of growth in *L. crispatus* and *G. vaginalis* discussed in this study offers important new insights and sets the stage for future research on bacterial metabolism.

## 5 Methods

**Bacterial culture:** The microbial strains utilized in this study were *Lactobacillus crispatus* DSM20584, *Gardnerella vaginalis* ATCC 14018, and *Lactobacillus iners* AB107 (ATCC 55195), all procured from the American Type Culture Collection (ATCC). *L. crispatus* was cultivated in de Man, Rogosa and Sharpe (MRS) medium for both planktonic and biofilm cultures. *G. vaginalis* planktonic cultures were propagated in NYCIII medium, while biofilm formation was induced using two distinct substrates: NYCIII (mentioned as Biofilm type 1 (B1)) and a modified Brain Heart Infusion (sBHI) medium supplemented with 2 % (w/v) gelatin, 0.5 % (w/v) yeast extract, and 0.1 % (w/v) soluble starch (mentioned as Biofilm type 2 (B2) throughout this paper). *L. iners* planktonic cultures were grown in Brain Heart Infusion (BHI) medium as they do not grow in MRS [48]. All cultures were incubated at 37 °C in a 5 % CO_2_, with planktonic cultures subjected to 24-h agitation at a speed of 100 rpm to avoid aggregation/biofilm formation and biofilm cultures were maintained under static conditions for 48 h. We used Hungate Anaerobic Tubes, 16 × 125 mm, (CLS-4208-01) to ensure minimal oxygen exposure to the cultures. All strains were confirmed by 16s rDNA PCR amplification using primers used elsewhere (Supplementary Fig. 2) [49,50].

**Preparation of cell-free culture supernatant:** All suspension cultures reached ∼0.8 OD_570_ in 24hrs. and filtered to obtain cell free supernatant after 24hrs. Supernatant from the biofilm were collected in around 48hrs followed by filtration. The same bacterial density was maintained for all cultures. cultures were centrifuged at 13000×*g* for 10mins and then sterile filtered using 0.2 μm filter. Cell-free supernatants were then subjected to non-targeted metabolomics in the Metabolon, NC, USA facility. Triplicates were used for each sample type.

**Non-targeted metabolomics:** The comprehensive metabolomic profiling was conducted externally by Metabolon, Inc. Briefly, the sample preparation process utilized the automated MicroLab STAR® system from Hamilton Company. Recovery standards were introduced before initiating the extraction for quality control and the resulting extract was separated into five fractions. Organic solvent removal was performed using a TurboVap® (Zymark). To ensure data quality and reliability, various control types were analyzed in parallel with the experimental samples. A pooled matrix sample generated by taking a small volume of each experimental served as a technical replicate throughout the data set; extracted water samples served as process blanks; and a cocktail of QC standards that were carefully chosen not to interfere with the measurement of endogenous compounds were spiked into every analyzed sample. This allowed performance monitoring and aided chromatographic alignment. UPLC-MS/MS was performed using a Waters ACQUITY UPLC platform interfaced with a Thermo Scientific Q-Exactive mass spectrometer, incorporating a HESI-II source and an Orbitrap analyzer with 35,000 mass resolution capability. Four distinct analytical methods were utilized as described by Metabolon. Metabolon's proprietary hardware and software which relies on a robust library of authenticated standards for compound identification was used for the process of data extraction, compound identification, and quality control. Metabolite quantification involved area-under-the-curve analysis of peaks. Data normalization was achieved by adjusting run-day block medians to 1.00 and scaling each data point accordingly. The metabolomics result was received with the list of metabolites and their intensity values for further analysis. We ran metabolomics analysis on the blank media conditions as a direct control for each bacterial culture. All cultures were normalized with the respective blank media to determine production and consumption ratios for each metabolite.

**Generation of heatmap:** The intensity values of each metabolite for all the microbes and culture conditions were normalized with the respective blank media used for culturing them. We have q-value and variance for each organism and culture condition for all identified metabolites (Supplementary file 1). We selected metabolites with p < 0.1 in comparison with media for further comparison.

log2 Fold change (FC) = log2 (Mean_B_/Mean_A_) (A is the blank media intensity value).

Log2 values were calculated and used to plot heatmaps using R studio (version 2022.12.0 + 353). To mitigate computational constraints and enhance visualization clarity, a threshold-based data reduction approach was implemented. Specifically, only metabolites exhibiting a log2 fold change magnitude of ≥4 (i.e., log2 FC ≥ 4 or ≤ −4) were selected for inclusion in the heatmap generation process, thereby focusing on metabolites with substantial differential abundance.

**Intra and inter-microbial comparison of metabolites between suspension and biofilm cultures:** Comparative metabolomic analysis was performed using Venn diagrams generated via the web-based tool at bioinformatics.psb.ugent.be to visualize the distribution of metabolites across *L. crispatus*, *G. vaginalis and L. iners* suspension and biofilm cultures (Only suspension culture for *L. iners*).

Metabolites unique to each intersection were subjected to metabolite set enrichment analysis (MSEA) using MetaboAnalyst 6.0 that used hypergeometric test to elucidate their respective metabolomic pathways. Pathway significance was visualized using bar diagrams created in GraphPad PRISM 10.0, with the y-axis representing -log10(p-value) for pathways meeting the significance threshold (p ≤ 0.059) (Supplementary Table 1). Box plots were constructed to depict the log2 fold change in intensity of statistically significant metabolites (p value ≤ 0.1), providing a quantitative representation of metabolite abundance variations across microbes and culture conditions (data submitted to Metabolomics Workbench).

## CRediT authorship contribution statement

**Smrutiti Jena:** Writing – review & editing, Writing – original draft, Software, Methodology, Investigation, Formal analysis, Data curation, Conceptualization. **Damilola Lawore:** Methodology, Data curation. **Leopold N. Green:** Writing – review & editing, Supervision. **Douglas K. Brubaker:** Writing – review & editing, Supervision, Resources, Project administration, Investigation, Funding acquisition, Conceptualization.

## Funding

This work is supported by startup funding from the Weldon School of Biomedical 10.13039/100000084Engineering, 10.13039/100006377Purdue University and 10.13039/100020950Department of Pathology at 10.13039/100008136Case Western Reserve University.

## Declaration of competing interest

The authors declare that they have no known competing financial interests or personal relationships that could have appeared to influence the work reported in this paper.

## Data Availability

The raw metabolomics data has been deposited in the Metabolomics Workbench under project number PR001854 and can be accessed via https://doi.org/10.21228/M82M8R.
