# Supplementary material for: Characterizing species-specific metabolic signatures in vaginal microbiota across planktonic and biofilm states
Source: Biofilm. 2025 Nov 13;10:100330. doi: 10.1016/j.bioflm.2025.100330 (PMC12682059; doi:10.1016/j.bioflm.2025.100330)
Supplement: Multimedia component 1 [file mmc1.pdf]

Fig S1: Suspension cultures of *L. iners* demonstrate distinctive metabolite profiles in terms of production and consumption, differing from *L. crispatus* and *G. vaginalis* cultures, with these metabolites linked to important metabolic pathways.

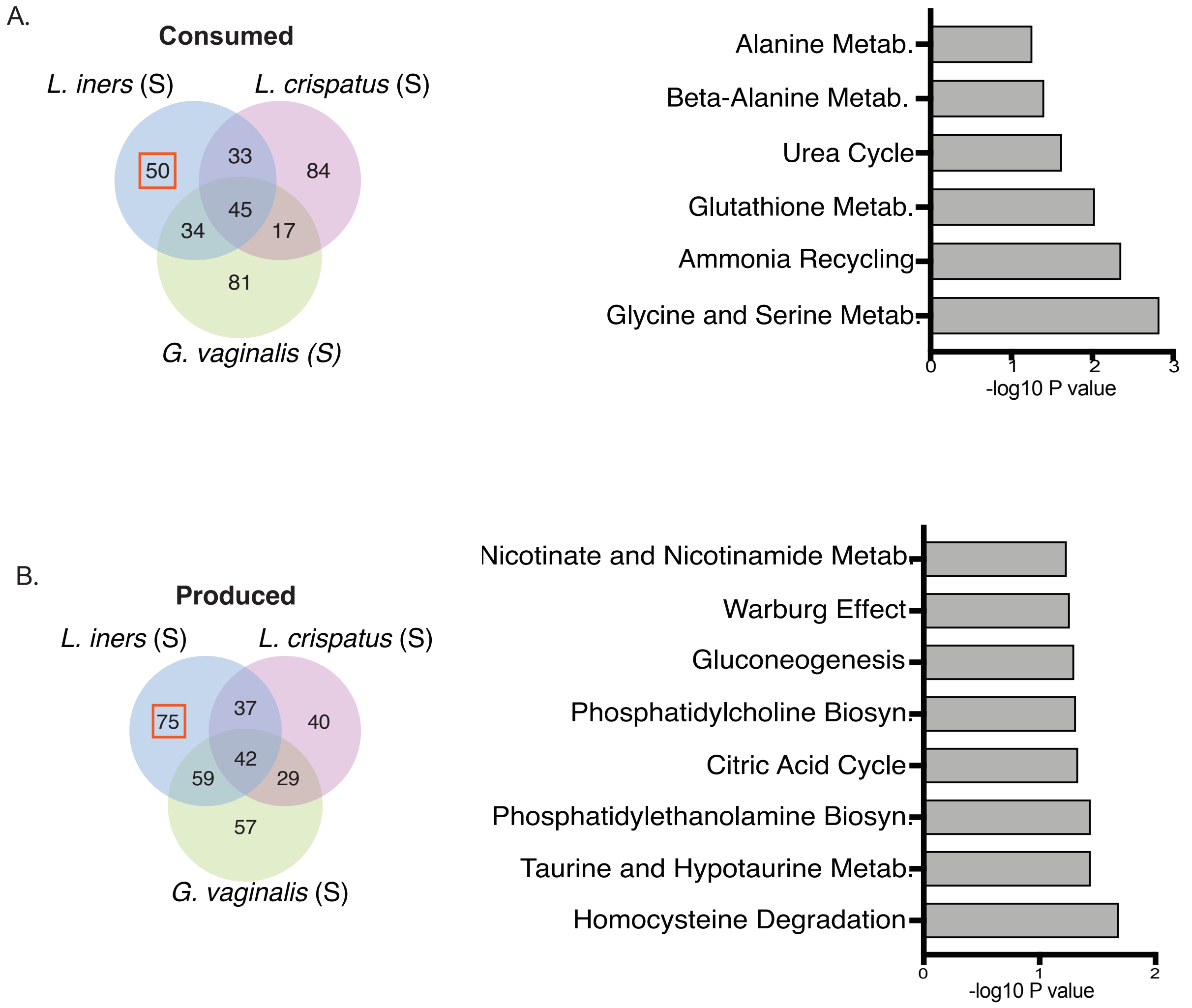

(A) Venn diagram showing distribution of consumed metabolites common or exclusive for suspension cultures of *L. iners, L. crispatus* and *G. vaginalis* (left), significant pathways for metabolites consumed exclusively by *L. iners* (right). (B) Venn diagram showing distribution of produced metabolites common or exclusive for suspension cultures of *L. iners, L. crispatus* and *G. vaginalis* (left), significant pathways for metabolites produced exclusively by *L. iners* (right).
Biological triplicate values were used to plot the graph. Included pathways with p value ≤ 0.059 and -log10 p value was used to plot the graphs. S: Suspension, Metab.: Metabolism, Biosyn.: Biosynthesis
