# Supplementary material for: Characterizing species-specific metabolic signatures in vaginal microbiota across planktonic and biofilm states
Source: Biofilm. 2025 Nov 13;10:100330. doi: 10.1016/j.bioflm.2025.100330 (PMC12682059; doi:10.1016/j.bioflm.2025.100330)
Supplement: Multimedia component 2 [file mmc2.pdf]

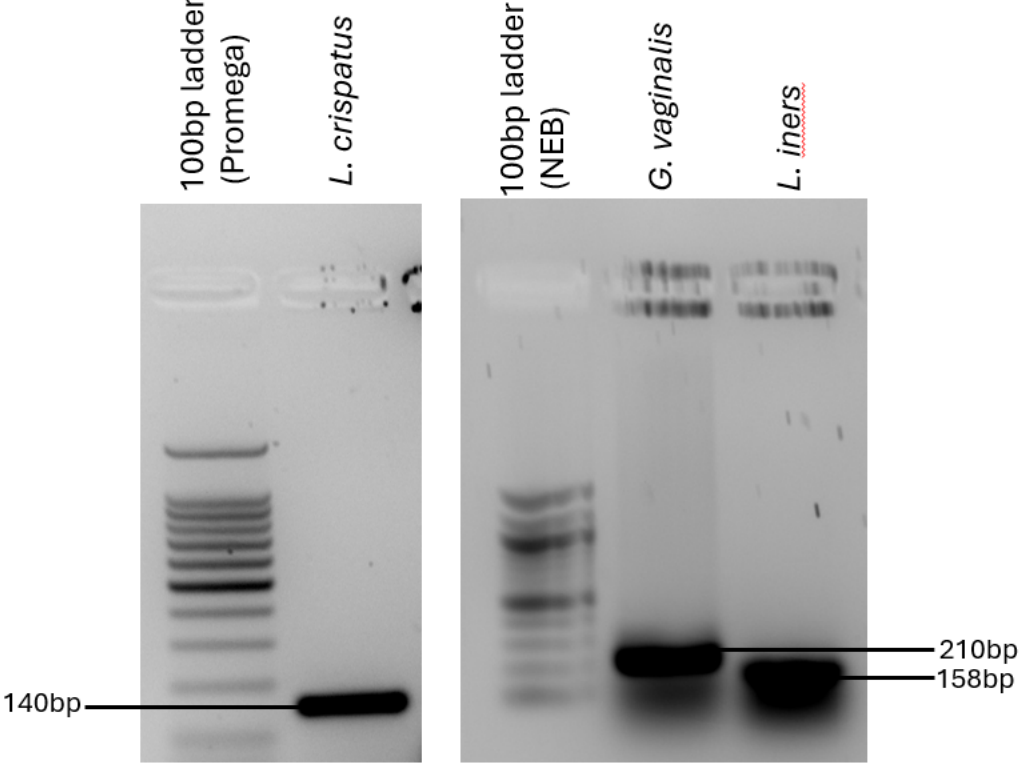

Supplementary Figure 2: Confirmation of bacterial strains using 16s gene PCR amplification. Lane 1; 100bp DNA ladder from Promega, Lane 2; 16s gene *L. crispatus*, Lane 3; 100bp DNA ladder from NEB, Lane 4; 16s gene *G. vaginalis* and Lane 5; *L. iners* 16s gene.
